# Impacts of Autophagy-Inducing Ingredient of Areca Nut on Tumor Cells

**DOI:** 10.1371/journal.pone.0128011

**Published:** 2015-05-27

**Authors:** Ching-Yu Yen, Wei-Fan Chiang, Shyun-Yeu Liu, Chung-Chih Lin, Kuo-An Liao, Che-Yi Lin, Wan-Fang Hsieh, Yon-Chi Cheng, Kai-Cheng Hsu, Pin-Yen Lin, Tai-Chi Chen, I-Ling Lee, Mei-Huei Lin, Young-Chau Liu

**Affiliations:** 1 Department of Dentistry, Taipei Medical University, Taipei, Taiwan; 2 Department of Dentistry, National Defense Medical Center, Taipei, Taiwan; 3 Oral and Maxillofacial Surgery Section, Chi Mei Medical Center, Tainan, Taiwan; 4 Oral and Maxillofacial Surgery Section, Chi Mei Hospital, Liouying, Taiwan; 5 School of Dentistry, National Yang-Ming University, Taipei, Taiwan; 6 Department of Life Sciences and Institute of Genome Sciences, National Yang-Ming University, Taipei, Taiwan; 7 Department of Biotechnology, Chia Nan University of Pharmacy, Tainan, Taiwan; 8 Department of Medical Research, Chi Mei Medical Center, Tainan, Taiwan; 9 Division of Natural Science, College of Liberal Education, Shu-Te University, Kaohsiung, Taiwan; University of Florida, UNITED STATES

## Abstract

Areca nut (AN) is a popular carcinogen used by about 0.6-1.2 billion people worldwide. Although AN contains apoptosis-inducing ingredients, we previously demonstrated that both AN extract (ANE) and its 30-100 kDa fraction (ANE 30-100K) predominantly induce autophagic cell death in both normal and malignant cells. In this study, we further explored the action mechanism of ANE 30-100K-induced autophagy (AIA) in Jurkat T lymphocytes and carcinoma cell lines including OECM-1 (mouth), CE81T/VGH (esophagus), SCC25 (tongue), and SCC-15 (tongue). The results showed that chemical- and small hairpin RNA (shRNA)-mediated inhibition of AMP-activated protein kinase (AMPK) resulted in the attenuation of AIA in Jurkat T but not in OECM-1 cells. Knockdown of Atg5 and Beclin 1 expressions ameliorated AIA in OECM-1/CE81T/VGH/Jurkat T and OECM-1/SCC25/SCC-15, respectively. Furthermore, ANE 30-100K could activate caspase-3 after inhibition of Beclin 1 expression in OECM-1/SCC25/SCC15 cells. Meanwhile, AMPK was demonstrated to be the upstream activator of the extracellular-regulated kinase (ERK) in Jurkat T cells, and inhibition of MEK attenuated AIA in Jurkat T/OECM-1/CE81T/VGH cells. Finally, we also found that multiple myeloma RPMI8226, lymphoma U937, and SCC15 cells survived from long-term non-cytotoxic ANE 30-100K treatment exhibited stronger resistance against serum deprivation through upregulated autophagy. Collectively, our studies indicate that Beclin-1 and Atg5 but not AMPK are commonly required for AIA, and MEK/ERK pathway is involved in AIA. Meanwhile, it is also suggested that long-term AN usage might increase the resistance of survived tumor cells against serum-limited conditions.

## Introduction

Macroautophagy (hereafter autophagy) is a conserved and homeostatic “self-eating” process involving lysosomal degradation of cytoplasmic components, which has been extensively studied in mammalian cells in the past two decades [1]. Impaired autophagy resulting in the inefficient removal of damaged organelles and cells may cause developmental abnormality, neurodegeneration, aging, inflammation, and cancer [2]. Thus, to harness autophagy in these diseases is now considered as a new strategy to improve human health [3]. Autophagy can either inhibit tumor formation by removing harmful materials or support the growth of established cancers by providing substrates for metabolism and maintaining the functional pool of mitochondria. It is thought that defining the context-specific role for autophagy in cancer and the involved mechanisms will be important to guide autophagy-based therapeutic intervention [4].

Most of the AuTophaGy-related (Atg) proteins cooperatively executing and regulating autophagy program are thought to have been identified [5]. However, accumulating evidences indicate that some key autophagy mediators can be dispensable and their mutual signaling positions along the pathway remain controversial. For example, AMP-activated protein kinase (AMPK), one of the central regulators of cellular metabolism in eukaryotes, is an energy sensor activated when intracellular ATP level decreases [6]. It serves as a relatively upstream regulator of autophagy and is activated by either LKB1 under low levels of glucose and O_2_ conditions or by calmodulin-dependent kinase kinase β (CAMKKβ) in response to intracellular calcium increase [7,8]. The requirement of AMPK for autophagy is shown by numerous evidences, however, autophagy can be executed in an AMPK-independent manner under conditions such as low glucose and ischemia/reperfusion [9,10]. Meanwhile, the relationship between AMPK and extracellular signal-regulated kinase (ERK) also remains elusive. AMPK can either inhibit or activate ERK [11–14], and interestingly, it can also be inhibited by ERK [15,16]. Likewise, another two essential autophagy mediators relatively downstream of AMPK such as Beclin 1 (the mammalian ortholog of yeast Atg6) and Atg5 can be excluded in some forms of autophagy [17–23]. Collectively, these features render the machineries of a certain type of autophagy unpredictable and some of them have been referred to as “alternative pathways” [24].

We have been investigating the impacts of the nut of *Areca catechu* L. (areca nut, AN) on cells. AN, a psychoactive and addictive carcinogen used by about 0.6–1.2 billion people around the world, contains the apoptosis-inducing ingredients including arecoline and oligomeric procyanidins [25,26]. Unexpectedly, we noticed that the crude extract of AN (ANE) and its 30–100 kDa fraction (named ANE 30–100K) can induce autophagic cell death in different cell lines and normal oral fibroblasts through reactive oxygen species [27,28]. Moreover, the autophagy-inducing activity of ANE 30–100K is sensitive to both cellulase and proteinase K suggesting the active ingredient to be a proteoglycan or glycoprotein [29]. Whether the ANE 30–100K-induced autophagy (AIA) represents a unique subtype of autophagy is currently unknown.

In this study, we tried to address the key players of AIA from conventional autophagy mediators such as AMPK, Beclin 1, and Atg5, as well as to delineate the relationship between AMPK and MEK/ERK along ANE 30–100K-mediated pathway. Because the epithelium of mouth and esophagus as well as infiltrated or circulating lymphocyte may encounter the stimulations of AN ingredients, we used different cell lines from these origins as the experimental models. On the other hand, we speculated that chronic ANE 30–100K stimulation might possibly elevate autophagic activity of tumor cells resulting in strengthened stress resistance. To simulate such physiological conditions, we used sublethal concentration of ANE 30–100K to stimulate several cell lines for 1–3 months and assessed whether autophagy activities in the survived cells are upregulated to increase their stress tolerance.

## Materials and Methods

### Cell culture and treatments

Oral epidermoid carcinoma OECM-1 [27] and esophageal carcinoma CE81T/VGH [28] cells were the kind gifts from Dr. Kuo-Wei Chang (Department of Dentistry, National Yang-Ming University, Taipei, Taiwan) and Dr. Cheng-Po Hu (Department of Medical Research and Education, Taipei Veterans General Hospital, Taipei, Taiwan), respectively. Leukemic Jurkat T [28], tongue carcinoma SCC15 [30] and SCC25 [30] were provided by Dr. Dar-Bin Shieh (Institute of Oral Medicine, National Cheng Kung University, Tainan, Taiwan). These five cells have been authenticated by short tandem repeat (STR) profiling by Bioresource Collection and Research Center (BCRC, Hsinchu, Taiwan). Lymphoma U937 (BCRC number 60435) and multiple myeloma RPMI8226 (BCRC number 60384) were purchased from BCRC. All the cells were expanded immediately after receipt and cryopreserved with multiple aliquots. Each aliquot was used within 6 months of resuscitation.

All the medium used contained 10% fetal bovine serum (FBS) (16000–044, Life Technologies Inc., Gibco/BRL Division, Grand Island, NY, USA) and cells were incubated at 37°C in a humid atmosphere with 5% CO_2_. Dulbecco’s modified Eagle’s medium (DMEM) (11995–065, Life Technologies Inc.) was used for OECM-1 and CE81T/VGH cells, RPMI 1640 medium (22400–105, Life Technologies Inc.) for Jurkat T, RPMI8226, and U937 cells, and 1:1 mixture of DMEM and Ham's F12 medium (11765–070, Life Technologies Inc.) containing 0.4 μg/ml hydrocortisone for SCC15 and SCC25 cells.

Carcinoma cells were seeded onto each well of a 96-well plate (5000 cells/well) for cell viability determination by XTT reagents (11465015001, Roche Molecular Biochemicals, Indianapolis, IN, USA) as instructed by the manufacturer, whereas suspended Jurkat, RPMI8226, U937 cells were 20,000 cells/well seeded and viable cell numbers were microscopically determined by trypan blue exclusion after treatment. 7 × 10^6^ carcinoma cells or 1.5 × 10^7^ suspended cells were seeded onto a 10-cm plate for Western blot analysis. Before these analyses, the cells were subjected to 24-hour serum starvation followed by treatment with each reagent under serum-free conditions for the indicated time periods.

### Preparation of ANE 30–100K

The preparation of ANE was described in our previous study [27]. Briefly, tender ANs were ground in a china bowl at room temperature, and the squeezed juice was centrifuged at 12,000g for 10 minutes at 4°C. The supernatant used as the ANE was further centrifuged at 2,900g for 30 minutes at 4°C with 30K- and 100K-pored membranous concentration tubes to collect the 30–100 kDa fraction. It was lyophilized and stored at -80°C. Upon usage, the dried powder was weighed and dissolved in H_2_O (designated as the ANE 30–100K).

### Western blot analysis

Lysate proteins (20 μg) from treated cells were subjected to Western blot analysis following the protocols previously described [27,28]. Briefly, the primary antibodies used include phosphor-Thr^172^ AMPKα (AMPK-p, 2535), total AMPKα (AMPK-T, 2603), and Atg5 (2630) from Cell Signaling Technology (Danvers, MA, USA); LC3 (L7543), Flag (F7425), and β-actin (A5441) from Sigma-Aldrich (St Louis, MO, USA); Beclin 1 (SC11427), pERK (SC-7383), and t-ERK (SC94) from Santa Cruz Biotechnology (Santa Cruz, CA, USA); and the secondary antibodies were horseradish peroxidase-coupled goat anti-mouse-IgG (AP124P) from Merck Millipore (Darmstadt, Germany) and goat anti-rabbit-IgG (81–6120) from Invitrogen Corporation (Camarillo, CA, USA).

### RNA interference

Bacteria containing shRNA sequences of AMPK and atg5 (Table 1), cloned into the *A*gel and *E*coRI restriction sites of pLKO.1-Puro plasmid, were purchased from the National RNAi Core Facility (NRCF, Academia Sinica, Taipei, Taiwan, as previously described [31]. After confirmation of these sequences, the constructed plasmids were packaged into lentivirus by NRCF and the multiplicity of infection (MOI) was determined. OECM-1, CE81T/VGH, SCC25, SCC15 cells (5–7 x 10^6^) and Jurkat T cells (1 x 10^7^) were cultured in medium containing plasmid-packaged virus (MOI = 4), polybrene (8 μg/ml) (Sigma-Aldrich, 107689), and 1% FBS for 24 hours. Cells were then selected in medium containing puromycin (2 μg/ml) and 10% FBS for 48 hours with or without further cloning, and cultured in 10% FBS-containing medium.

**Table 1:** shRNA constructs used in this study.

Target sequence	Oligo sequence	Gene reference
sh-AMPK CDS	CCGGCCTGGAAGTCACACAATAGAACTC	NM_006251.5 (1621–1641 bp)
CCTGGAAGTCACACAATAGAA	GAGTTCTATTGTGTGACTTCCAGGTTTTT^1^	http://www.ncbi.nlm.nih.gov/nuccore/NM_006251.5
sh-AMPK 3’UTR	CCGGGCATAATAAGTCACAGCCAAACTC	NM_006251.5 (1726–1746 bp)
GCATAATAAGTCACAGCCAAA	GAGTTTGGCTGTGACTTATTATGCTTTTT	http://www.ncbi.nlm.nih.gov/nuccore/NM_006251.5
sh-atg5 CDS	CCGGCCTTTCATTCAGAAGCTGTTTCTC	NM_004849.2 (942–962 bp)
CCTTTCATTCAGAAGCTGTTT	GAGAAACAGCTTCTGAATGAAAGGTTTTT	http://www.ncbi.nlm.nih.gov/nuccore/92859692
sh-beclin 1	CGGGCCAGACAGATGTGGATTTCAAGAGA	NM_003766.3 (542–561 bp)
GGGCCAGACAGATGTGGAT	ATCCACATCTGTCTGGCCCTTTTTGGAAA	http://www.ncbi.nlm.nih.gov/nuccore/NM_003766.3

^1^The complementary sequences of shRNA were underlined.

Alternatively, the synthetic beclin 1 shRNA (Table 1) was cloned into the EcoRI and HindIII restriction sites of pHsU6 plasmid, a kind gift from Dr. Ming-Derg Lai (Institute of Medical Basic Sciences of National Cheng Kung University, Tainan, Taiwan), and the sequences were also further confirmed. OECM-1 cells (1 × 10^6^) were seeded onto 6-cm dish for two days and then cells were cultured in 0.4 ml Opti-MEM (31985–088, Life Technologies) and transfected with empty or beclin1 shRNA-inserted plasmid by Lipofectamine (L3000-015, Invitrogen) (1:1) as instructed by the manufacturer. Cells were cultured in DMEM containing 20% FBS for 48 hours and subjected to puromycin (2 μg/ml) selection and cloning.

### Puncta analysis

The green fluorescent protein-LC3 (GFP-LC3) construct was bought from Addgene (plasmid 11546, Cambridge, MA, USA). Lipofectamine-mediated GFP-LC3 transfection into Jurkat T (1 × 10^7^), OECM-1 (1 × 10^6^), and SCC25 (1 × 10^6^) cells was basically performed as the previous section, except that Jurkat T cells were cultured in RPMI 1640 medium throughout this process. All the three cells were selected by puromycin (2 μg/ml) for 3–5 days. OECM-1 or SCC25 (1 × 10^4^) cells were seeded into each well of a 10-well chamber slide, whereas Jurkat T cells (5 × 10^4^) were introduced into each eppendorf tube, in 0.2 ml medium. OECM-1 or SCC25 cells were then subjected to the indicated treatment and washed with cold PBS, fixed with 3.7%formaldehyde/PBS at 4°C for 20 minutes, washed twice with PBS, and visualized under a laser-scanning confocal microscope (Olympus FV1000). The washing and fixing processes of Jurkat T cells were performed in eppendorf tubes and then placed on a slide. The excess medium on the slide was air-dried in a fume hood, and cells were then observed under the Olympus FV1000 microscope.

### Electroporation of AMPK expression vector

Cloned sh-AMPK 3’UTR-A1 (Jurkat T) cells were pelleted and resuspended in 100 μl transfection buffer at a concentration of 6 × 10^5^ cells/12 μl and then transferred to a sterile 3-mm Amaxa nucleofection cuvette. Cells were incubated with 2.4 μg AMPK expression vector, pWZL Neo Myr Flag PRKAA1 (plasmid 20595, Addgene), and electroporated with pulse voltage: 1 V, pulse width: 30 milliseconds, and pulse number: 2 with Microporator MP-100 (Digital Bio Technology, Seoul, South Korea) as instructed by the User’s Manual. Cells were then rinsed with 500 μl of sterile culture medium and transferred to the well of a sterile 12-well plate. Cells were incubated at 37°C for 24 hours before neomycin (200 μg/ml) selection and cloning.

### Observation and quantification of acidic vesicle-containing cells

Treated cells were stained with acridine orange (1 μg/ml) for 10 minutes and photographed under a fluorescent microscope as described earlier [27]. The percentage of acidic vesicle-containing cells in randomly chosen 100 cells from three independent microscopic fields was determined.

### Caspase-3 activity assay

7 × 10^6^ parental and Beclin 1-knocked down OECM-1, SCC25, and SCC15 cells seeded onto 10-cm dishes were serum starved overnight and treated with ANE 30–100K (7.5 μg/ml) for 24 hours under SF conditions. Cell lysates were prepared and 50 μg lysate proteins were subjected to the analysis by Caspase 3 Assay Kit, Colorimetric (CASP3C, Sigma-Aldrich) in a triplicated manner as instructed. The reaction for color development was held at 37°C for 90 minutes, and the value of OD_405_ was determined.

### Long-term ANE 30–100K stimulation of three different cells

To obtain long-term ANE 30–100K-stimulated cells, multiple myeloma RPMI8226, lymphoma U937, and tongue carcinoma SCC15 cells were cultured in medium containing ANE 30–100K (1.25 μg/ml) and 10% FBS for 30–90 days. The survived cells were then subjected to serum starvation with or without the presence of 3-MA (1 μM) and CQ (25 μM) for 24 hours, followed by analyses of cell viability and LC3/β-actin ratio by XTT and Western blot assays, respectively.

### Detection of DNA fragmentation

5 × 10^6^ OECM-1 cells treated with the indicated concentrations of AN ingredients for 24 hours were washed twice with PBS and fixed with 1 ml ethanol (70%). After storage overnight at 4°C, the ethanol was removed and the cells were resuspended in 1 ml phosphate-citric acid buffer with 0.2 M Na_2_HPO_4_ and 0.1 M citric acid (pH 7.8) at room temperature for 60 minutes with occasional shaking. The cell suspension was centrifuged at 2000 rpm for 5 minutes. The supernatant containing low molecular mass DNA was collected for analysis of internucleosomal DNA degradation by agarose gel electrophoresis.

### Statistical analysis

Two groups of data presented as mean ± SD were analyzed by Student’s *t*-test. A value of *P* < 0.05 was regarded as statistically significant.

## Results

### ANE 30–100K is an autophagy inducer and phosphorylates AMPKα-Thr^172^

**Fig 1:**
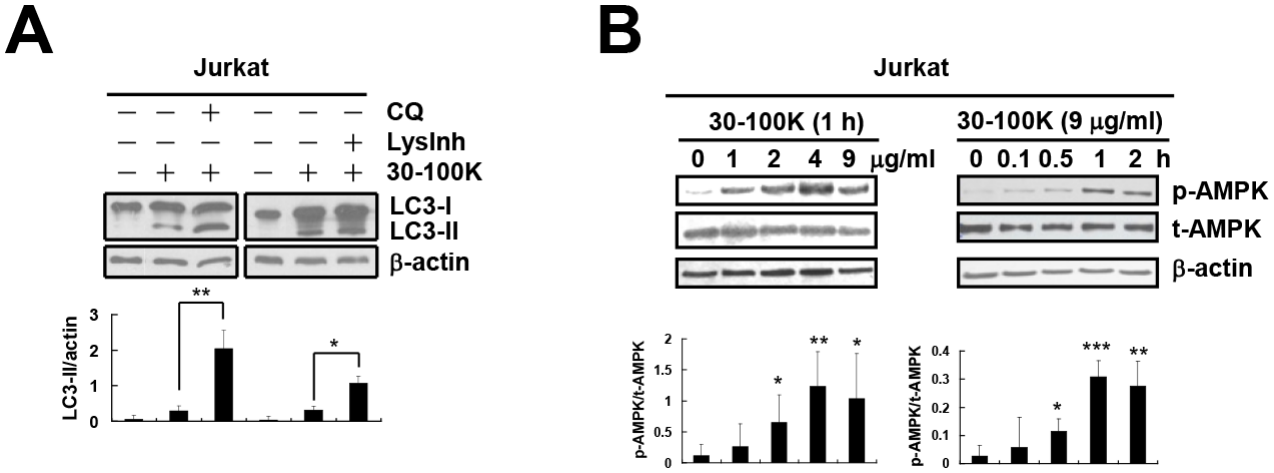
ANE 30–100K induces autophagic flux and AMPKα-Thr^172^ phosphorylation in Jurkat T cells. (A) Immunoblot demonstrating LC3 and β-actin proteins of Jurkat T cells treated with or without ANE 30–100K (30–100K, 15 μg/ml), chloroquine (CQ, 30 μM) and lysosomal inhibitors (LysInh, pepstatin A 10 μg/ml, E64d 10 μg/ml, and leupeptin 10 μg/ml) as indicated. The average LC3-II/actin ratio ± SD from three independent experiments were plotted under each lane. (B) Lysates of Jurkat T cells treated with 30–100K (0–9 μg/ml) for 1 hour (left) or 30–100K (9 μg/ml) for 0–24 hours (right) were immunoblotted with p-AMPK, t-AMPK, and β-actin antibodies. Average p-AMPK/t-AMPK ratio ± SD from three independent experiments were plotted under each lane. *P < 0.05, **P < 0.01, ***P < 0.001.

We previously showed the induction of autophagic flux by ANE 30–100K in normal oral fibroblasts and esophageal carcinoma CE81T/VGH cells [28]. Here, we demonstrated that both autophagy inhibitors, the chloroquine (CQ) and cocktail of lysosomal inhibitors (LysInh, containing pepstatin A, E64D, and leupeptin), further increased ANE 30–100K-induced elevation of LC3-II level in Jurkat T cells (Fig 1A). Similar results were also observed in oral carcinoma OECM-1 cells (S1A Fig). These data suggested ANE 30–100K as an autophagy inducer in different types of cancer cells.

We firstly speculated AMPK as a potential mediator of AIA and demonstrated the concentration- and time-dependent induction of AMPKα-Thr^172^ phosphorylation by ANE 30–100K in Jurkat T cells (Fig 1B) and CE81T/VGH (S1B Fig).

### Chemical inhibitors of AMPK and CAMKKβ attenuate AIA in Jurkat T cells

**Fig 2:**
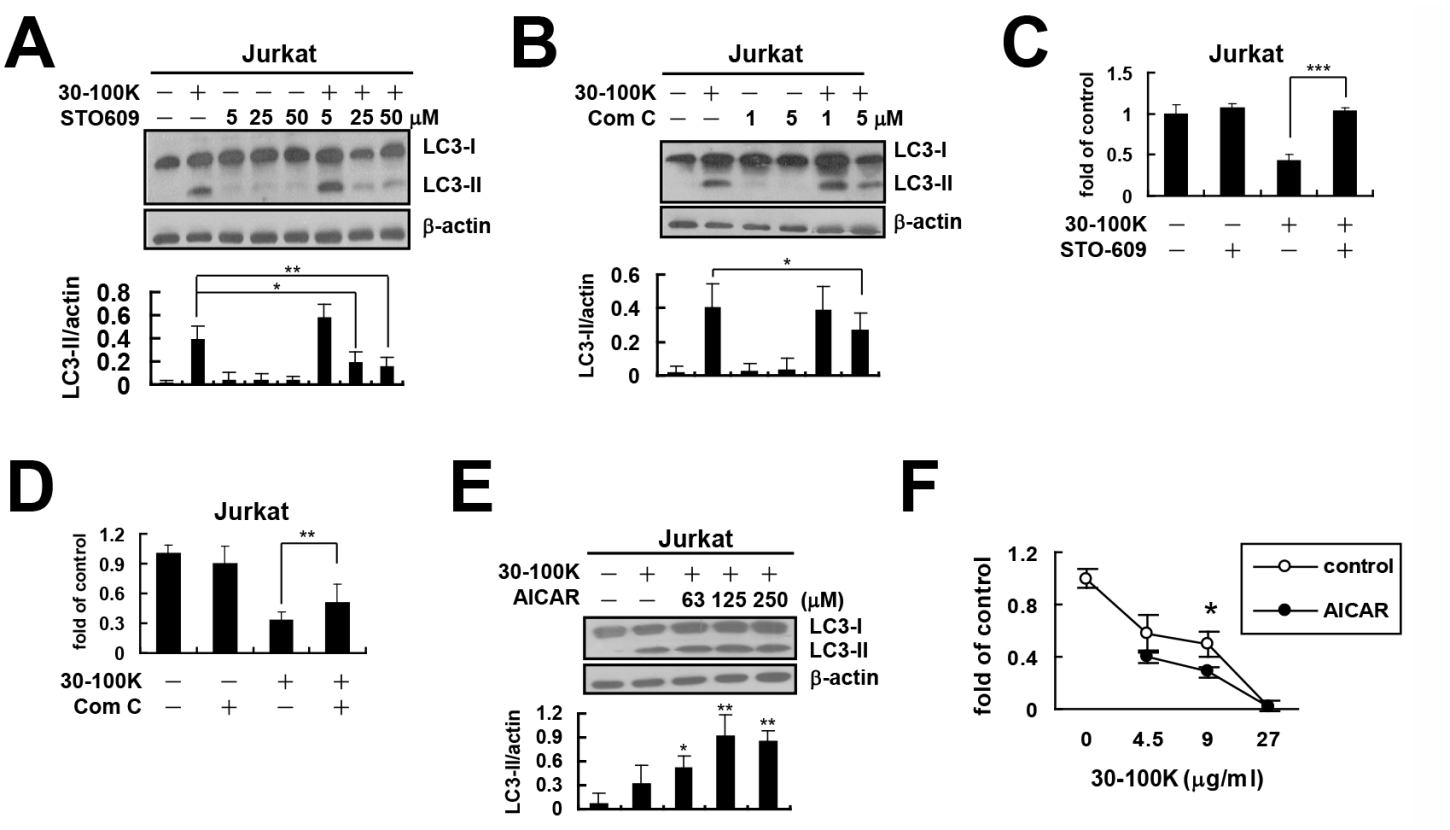
Chemical inhibitors of AMPK and CAMKKβ attenuate AIA. Jurkat T cells treated with ANE 30–100K (30–100K) (9 μg/ml) for 24 hours in the presence or absence of STO-609 (50 μM) (A) or compound C (Com C, 5 μM) (B) were subjected to LC3 and β-actin immunoblotting. Average LC3-II/actin ratio ± SD were plotted as Fig 1A. Cell numbers of Jurkat T cells pretreated with STO-609 (50 μM) (C) or Com C (5 μM) (D) for 2 hours, followed by 30–100K (9 μg/ml) treatment for another day were counted and average fold of untreated control cells ± SD from three independent experiments were plotted. (E) Lysates of Jurkat T cells treated with 30–100K (9 μg/ml) for 24 hours in the presence or absence of the indicated concentrations of AICAR were immunoblotted and LC3-II/actin ratios were presented as (A). (F) Cell numbers of Jurkat T cells treated with the indicated concentrations of 30–100K for 24 hours in the presence or absence of AICAR (250 μM) were determined and average fold of untreated control cells ± SD were plotted. *P < 0.05, **P < 0.01, ***P < 0.001.

We next assessed the role of AMPK in AIA by chemical inhibitors. Both STO-609 (CAMKKβ inhibitor) and compound C (AMPK inhibitor) were demonstrated to ameliorate ANE 30–100K-induced LC3-II accumulation (Fig 2A and 2B, respectively) and cytotoxicity (Fig 2C and 2D, respectively) in Jurkat cells.

We also used the AMPK activator, 5-aminoimidazole-4-carboxamide ribonucleoside (AICAR) to assess whether it can enhance the effects of ANE 30–100K. The results showed that AICAR dose-dependently increased ANE 30–100K-induced LC3-II accumulation (Fig 2E) and cytotoxicity (Fig 2F) in Jurkat cells. These results suggested the involvement of CAMKKβ/AMPK pathway in AIA of Jurkat T cells.

### Inhibition of AMPK expression attenuates AIA in Jurkat T but not in OECM-1 cells

**Fig 3:**
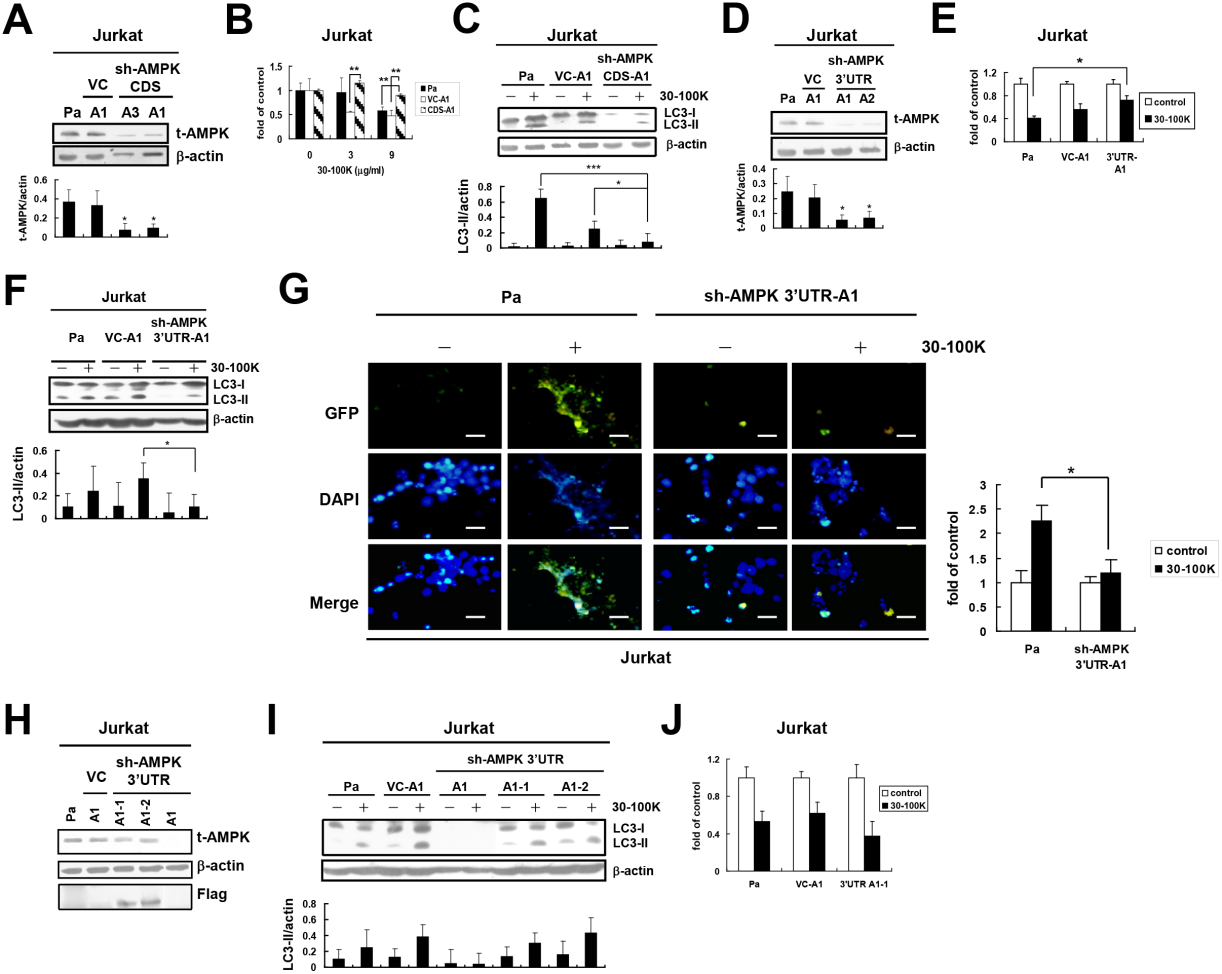
AMPK is required for AIA in Jurkat T cells. (A) Parental (Pa) Jurkat T cells were transduced with empty pLKO.1-Puro plasmid (virus control, VC) or AMPK coding sequence (CDS)-shRNA-pLKO.1-Puro plasmid followed by puromycin selection and cloning. Lysates of Pa, VC-A1 clone, and two AMPK-knocked down clones (sh-AMPK CDS-A3 and CDS-A1) were immunoblotted with total AMPK (t-AMPK) and β-actin antibodies. Average t-AMPK/actin ratio ± SD from three representative experiments were plotted under each lane. (B) Cell numbers of Pa, VC-A1, and sh-AMPK CDS-A1 after treatment with the indicated concentrations of ANE 30–100K (30–100K) for 24 hours were determined and presented as Fig 2C. (C) Lysates of Pa, VC-A1, and sh-AMPK CDS-A1 cells treated with or without 30–100K (9 μg/ml) for 24 hours were immunoblotted and presented as Fig 1A. (D) Pa Jurkat T cells were also infected with the same plasmid containing AMPK 3’-untranslated region (3’UTR) and two representative clones sh-AMPK 3’UTR-A1 and 3’UTR-A2 were generated. Pa, VC-A1, and sh-AMPK 3’UTR-A1 and 3’UTR-A2 cells were subjected to the same immunoblot and the data were presented as (A). (E) The sensitivity of Pa, VC-A1, and sh-AMPK 3’UTR-A1 cells to ANE 30–100K (9 μg/ml) was accessed as (B). (F) Pa, VC-A1, and sh-AMPK 3’UTR-A1 cells were subjected to the same treatment and immunoblot as (C). (G) Pa and sh-AMPK 3’UTR-A1 cells electroporated with LC3-GFP construct and treated with or without 30–100K (9 μg/ml) were photographed under a fluorescent microscope. The percentage of puncta-containing cells were determined in randomly chosen 200 cells and average fold of untreated control cells ± SD were plotted. Bar = 10 μm. (H) sh-AMPK 3’UTR-A1 clone was further electroporated with full-length AMPK and Flag-tagged expression vector, and subjected to neomycin selection and cloning. Two representative sh-AMPK 3’UTR-A1-1 and 3’UTR-A1-2 clones were obtained. Lysates of Pa, VC-A1, 3’UTR-A1-1, 3’UTR-A1-2, and 3’UTR-A1 cells were immunoblotted with t-AMPK, β-actin, and Flag antibodies. (I) Induction of LC3-II accumulation in these five cells treated with or without 30–100K (9 μg/ml) was immunoblotted and presented as (C). (J) The sensitivity of Pa, VC-A1, and sh-AMPK 3’UTR-A1-1 cells against 30–100K (9 μg/ml) was assessed and presented as (E). *P < 0.05, **P < 0.01.

Since AICAR and compound C can mediate AMPK-independent cellular responses [32,33], we further tried to downregulate AMPK expression by the lentivirus-mediated shRNA interference to analyze AMPK’s role in AIA. Firstly, viruses carrying an AMPK shRNA fragment located at the coding sequence (CDS) of AMPK gene (Table 1) were used to infect Jurkat T cells followed by puromycin selection and cloning. The results showed that compared to parental (Pa) Jurkat T cells and virus control cells (VC-A1 clone, infected by viruses containing empty plasmid and cloned by puromycin), AMPK expressions in AMPK shRNA-transduced (sh-AMPK) CDS-A1 and CDS-A3 clones were profoundly inhibited (Fig 3A). Furthermore, sh-AMPK CDS-A1 cells became more resistant to cytotoxic ANE 30–100K challenge and generated a lower LC3-II level than those of Pa and VC-A1 cells after ANE 30–100K treatment (Fig 3B and 3C, respectively). Moreover, another AMPK shRNA fragment corresponding to the 3’ untranslated region (3’UTR) of AMPK gene (Table 1) was also used to knock down AMPK expression. Two clones (sh-AMPK 3’UTR-A1 and 3’UTR-A2) receiving prominent inhibitory effect on AMPK expression were obtained (Fig 3D). The sh-AMPK 3’UTR-A1 clone was also more tolerable to cytotoxic ANE 30–100K and expressed a lower level of LC3-II protein than those of Pa and VC-A1 cells (Fig 3E and 3F, respectively). Furthermore, ANE 30–100K-stimulated generation of GFP-LC3 puncta was significantly reduced in sh-AMPK 3’UTR-A1 clone than that in Pa cells (Fig 3G).

Next, the rescue of AMPK expression in sh-AMPK 3’UTR-A1 cells by electroporation with flag-tagged AMPK expression construct was applied to analyze whether AIA can be restored. Two clones (sh-AMPK 3’UTR A1-1 and 3’UTR A1-2) with recovered AMPK expression were obtained (Fig 3H). The induction effect of ANE 30–100K on LC3-II level was resumed in these two clones (Fig 3I), and the sh-AMPK 3’UTR A1-1 clone became more sensitive to cytotoxic ANE 30–100K challenge (Fig 3J). These data collectively indicated the requirement of AMPK for AIA in Jurkat T cells.

**Fig 4:**
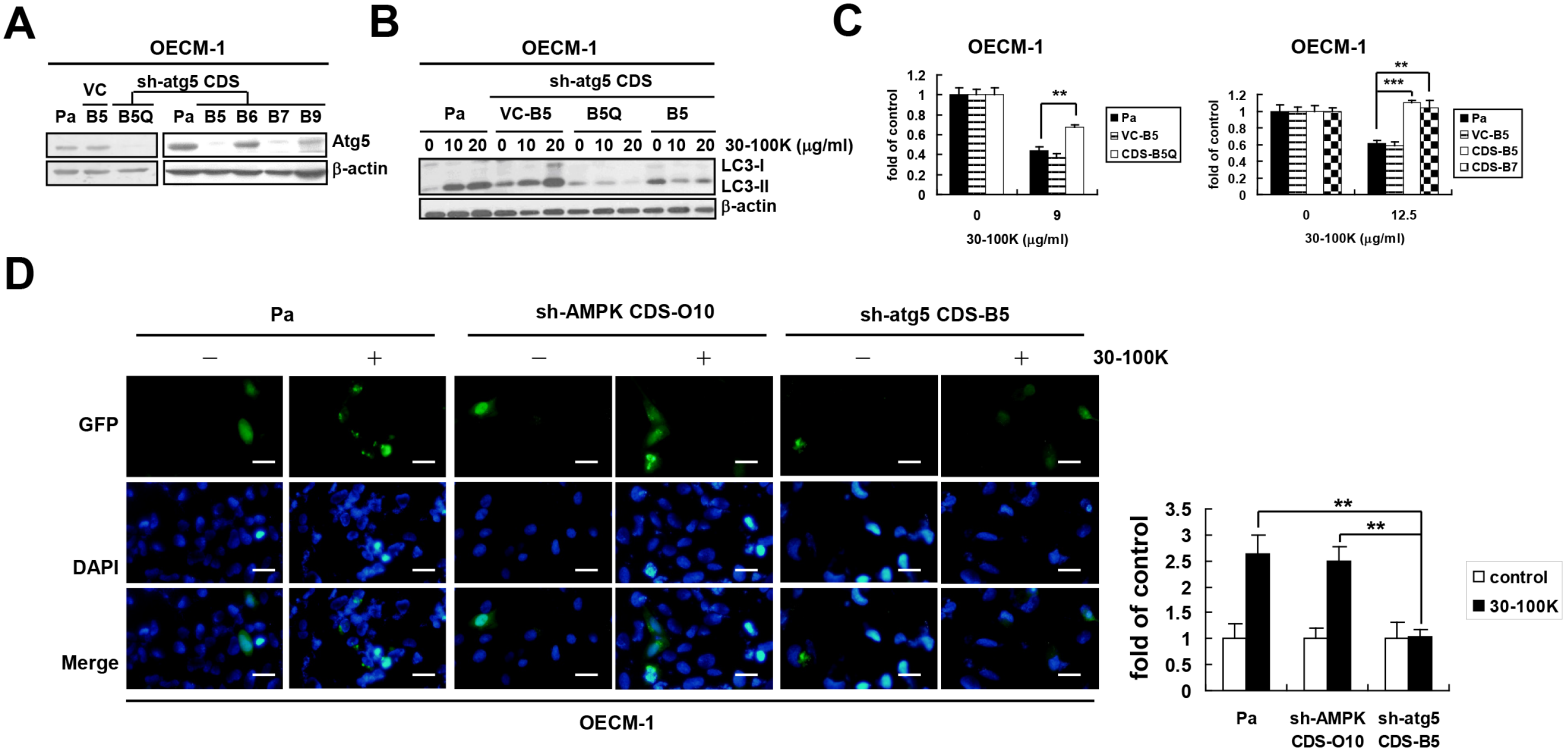
Atg5 is required for AIA in OECM-1 cells. (A) Atg5 was knocked down in OECM-1 by using the same strategy as Fig 3. Lysates of parental (Pa), virus control (VC-B5 clone), and cloned sh-atg5 CDS-BQ5, CDS-B5, CDS-B6, CDS-B7, and CDS-B9 OECM-1 cells were immunoblotted with Atg5 and β-actin antibodies. (B) Induction of LC3-II by ANE 30–100K (30–100K, 0, 10, or 20 μg/ml) in Pa, VC-B5, sh-atg5 CDS-B5Q, and CDS-B5 cells were analyzed as Fig 1A. (C) Viability of Pa, VC-B5, and sh-atg5 CDS-B5Q cells (left), as well as CDS-B5 and CDS-B7 cells (right), treated with or without 30–100K (15 μg/ml) was assessed by XTT. Average OD_450_ values against those of untreated control cells ± SD were plotted. (D) Pa, sh-AMPK CDS-O10, and sh-atg5 CDS-B5 OECM-1 cells electroporated with LC3-GFP construct and treated with or without 30–100K (15 μg/ml) were photographed under a fluorescent microscope. The percentage of puncta-containing cells was determined and presented as Fig 3G. Bar = 10 μm. **P < 0.01, ***P < 0.001.

In contrast to the role of AMPK in AIA of Jurkat T cells, we found that the phosphorylation level of AMPKα-Thr^172^ in oral carcinoma OECM-1 cells was not further increased by ANE 30–100K (S2A Fig). Neither STO-609 nor compound C protected OECM-1 cells from the cytotoxic insult of ANE 30–100K (S2B Fig). Three representative AMPK-knocked down OECM-1 clones (sh-AMPK CDS-O8, CDS-O9, and CDS-O10) transduced with the same AMPK shRNA as Fig 3A were generated (S2C Fig) and responded similarly to cytotoxic ANE 30–100K treatment as Pa and virus control (VC-A5 clone) cells (S2D Fig). In addition, ANE 30–100K induced similar patterns of concentration-dependent LC3-II accumulation in OECM-1 VC-A5 and CDS-O10 clones (S2E Fig) as well as comparable percentages of puncta-containing cells in CDS-O10 and Pa cells (Fig 4D). These evidences suggested that AMPK may be redundant for AIA in OECM-1 cells.

### Atg5 is required for AIA in OECM-1, CE81T/VGH, and Jurkat T cells

We continued to search for other mediators of AIA in OECM-1 cells and firstly chose Atg5 as the potential target. By using the same strategy with the atg5 CDS shRNA fragment (Table 1), three representative Atg5-downregulated OECM-1 clones (sh-atg5 CDS-B5Q, CDS-B5, and CDS-B7) were generated (Fig 4A). ANE 30–100K concentration-dependently induced LC3-II accumulation in Pa and virus control (VC-B5 clone) cells but not in CDS-B5Q and CDS-B5 clones (Fig 4B). CDS-B5Q, CDS-B5, and CDS-B7 cells were more tolerable to cytotoxic ANE 30–100K challenge (Fig 4C, left and right). Furthermore, after ANE 30–100K treatment, sh-atg5 CDS-B5 showed a lower percentage of GFP-LC3 puncta-containing cells than that of Pa cells (Fig 4D). These results suggested an essential role of Atg5 for AIA in OECM-1 cells.

The role of Atg5 in AIA was also analyzed in CE81T/VGH and Jurkat T cells. By using the same methods, sh-atg5 CDS-A3 and CDS-A5 clones of CE81T/VGH cells were acquired with mildly and profoundly inhibited Atg5 expression, respectively (S3A Fig). CDS-A5 clone was more resistant to the cytotoxicity of ANE 30–100K than Pa, virus control (VC-A3 and VC-A4 clones), and CDS-A3 cells (S3B Fig) and produced lower level of acidic vesicle (AV)-containing cells than those of Pa, VC-A3, and VC-A4 cells after ANE 30–100K treatment (S3C Fig). Furthermore, unlike that in VC-A4 clone, ANE 30–100K-induced LC3-II increase in CDS-A5 clone was abolished (S3D Fig).

In the case of Jurkat T cells, the expression level of Atg5 was barely detectable after transduction of sh-atg5 shRNA without further cloning (S3E Fig). These cells also became more resistant to ANE 30–100K’s cytotoxicity than Pa cells (S3F Fig). Collectively, Atg5 is suggested to be a common mediator of AIA among different cells.

### Beclin 1 is required for AIA in OECM-1, SCC25, and SCC15 cells

**Fig 5:**
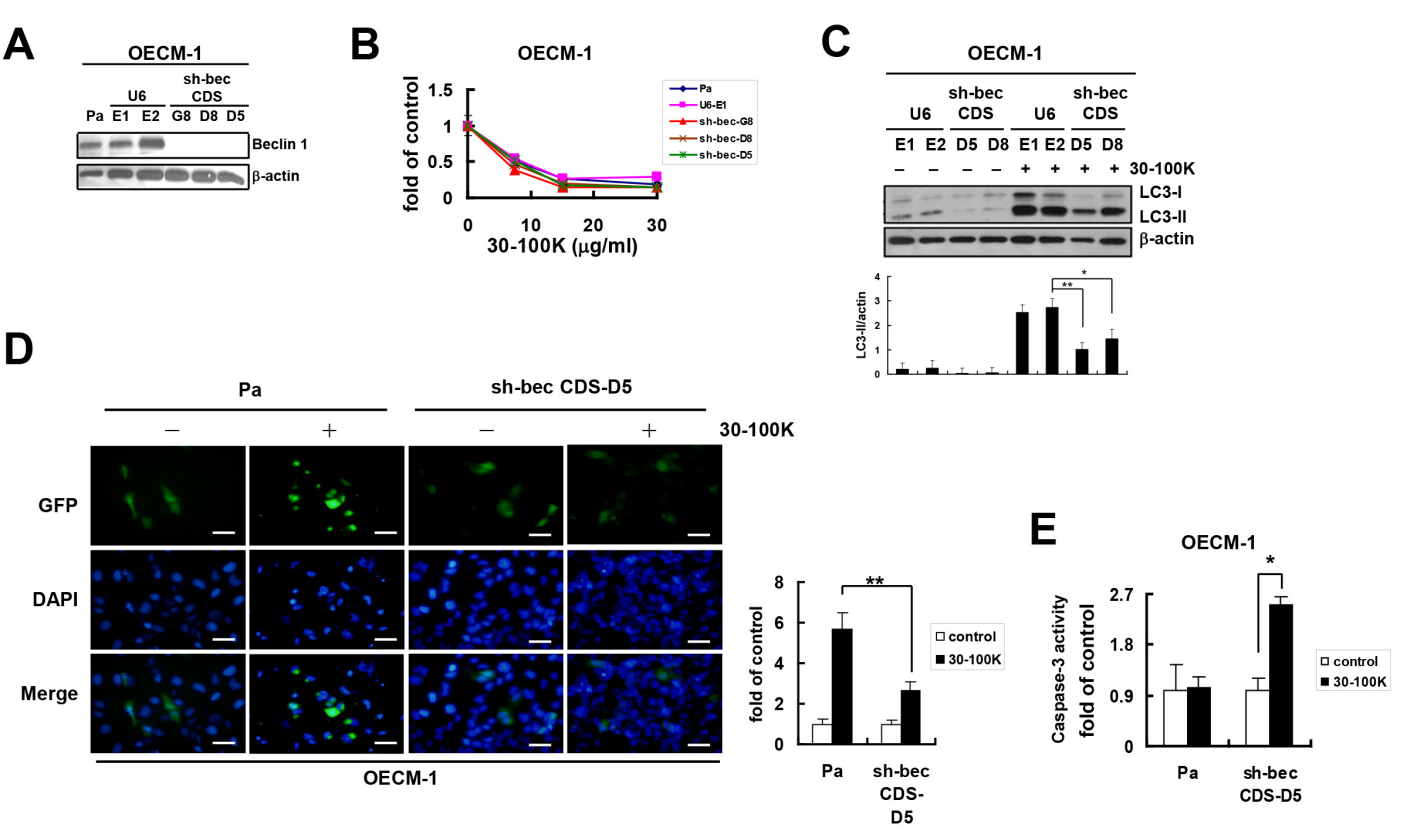
Beclin 1 knockdown inhibits AIA and activates caspases-3 in OECM-1 cells. (A) Parental (Pa) OECM-1 cells transfected with either empty pHsU6 plasmid (U6) or beclin 1 coding sequence (CDS) shRNA-pHsU6 construct were subjected to puromycin selection and cloning. Lysates of Pa cells, plasmid control clones (U6-E1 and U6-E2), and Beclin 1-knocked down clones (sh-bec CDS-G8, CDS-D8, and CDS-D5) were immunoblotted with Beclin 1 and β-actin antibodies. (B) Pa, U6-E1, sh-bec CDS-G8, CDS-D8, and CDS-D5) cells treated with ANE 30–100K (30–100K, 0, 7.5, 15, and 30 μg/ml) for 24 hours were subjected to XTT assay and presented as Fig 4C. (C) Induction of LC3-II in U6-E1, U6-E2, sh-bec CDS-D5, and CDS-D8 cells by 24-h 30–100K (15 μg/ml) treatment was analyzed and presented as Fig 1A. (D) Pa and sh-bec CDS-D5 cells transfected with LC3-GFP construct and treated with or without 30–100K (15 μg/ml) were photographed and presented as Fig 3G. Bar = 10 μm. (E) Caspase-3 activity in Pa and sh-bec CDS-D5 OECM-1 cells treated with 30–100K (15 μg/ml) for 24 hours were assessed by the colorimetric kits. Average OD_405_ absorbances against untreated control ± SD were plotted. *P < 0.05, **P < 0.01.

It was speculated that another core autophagy mediator Beclin 1 could play a role in AIA. By transfecting cells with pHsU6 plasmid containing synthetic beclin 1 shRNA fragment (Table 1), three representative sh-bec CDS-G8, CDS-D8, and CDS-D5 clones of OECM-1 expressing undetectable Beclin 1 protein were obtained (compared to Pa and two empty plasmid-transfected control U6-E1 and U6-E2 clones) (Fig 5A). Unexpectedly, these three clones exhibited similar sensitivity against cytotoxic ANE 30–100K insult as those of Pa and U6-E1 cells (Fig 5B). It was originally thought that Beclin 1 might be redundant for AIA. However, ANE 30–100K was then shown to induce lower levels of LC3-II protein in sh-bec CDS-D5 and CDS-D8 clones than those in U6-E1 and U6-E2 clones (Fig 5C). In addition, the percentage of ANE 30–100K-induced GFP-LC3 puncta-containing cells (Fig 5D) was significantly lower in sh-bec CDS-D5 clone than that in Pa cells. These results suggested the requirement of Beclin 1 for AIA in OECM-1.

Since Beclin 1 is involved in the regulation of both autophagy and apoptosis, and autophagy inhibition may lead to apoptosis activation [34], it is speculated that the lack of protection effect of Beclin 1 knockdown on cytotoxic ANE 30–100K was due to activation of apoptotic program. Indeed, ANE 30–100K was verified to stimulate caspase-3 activation in sh-bec CDS-D5 OECM-1 but in not Pa cells (Fig 5E).

**Fig 6:**
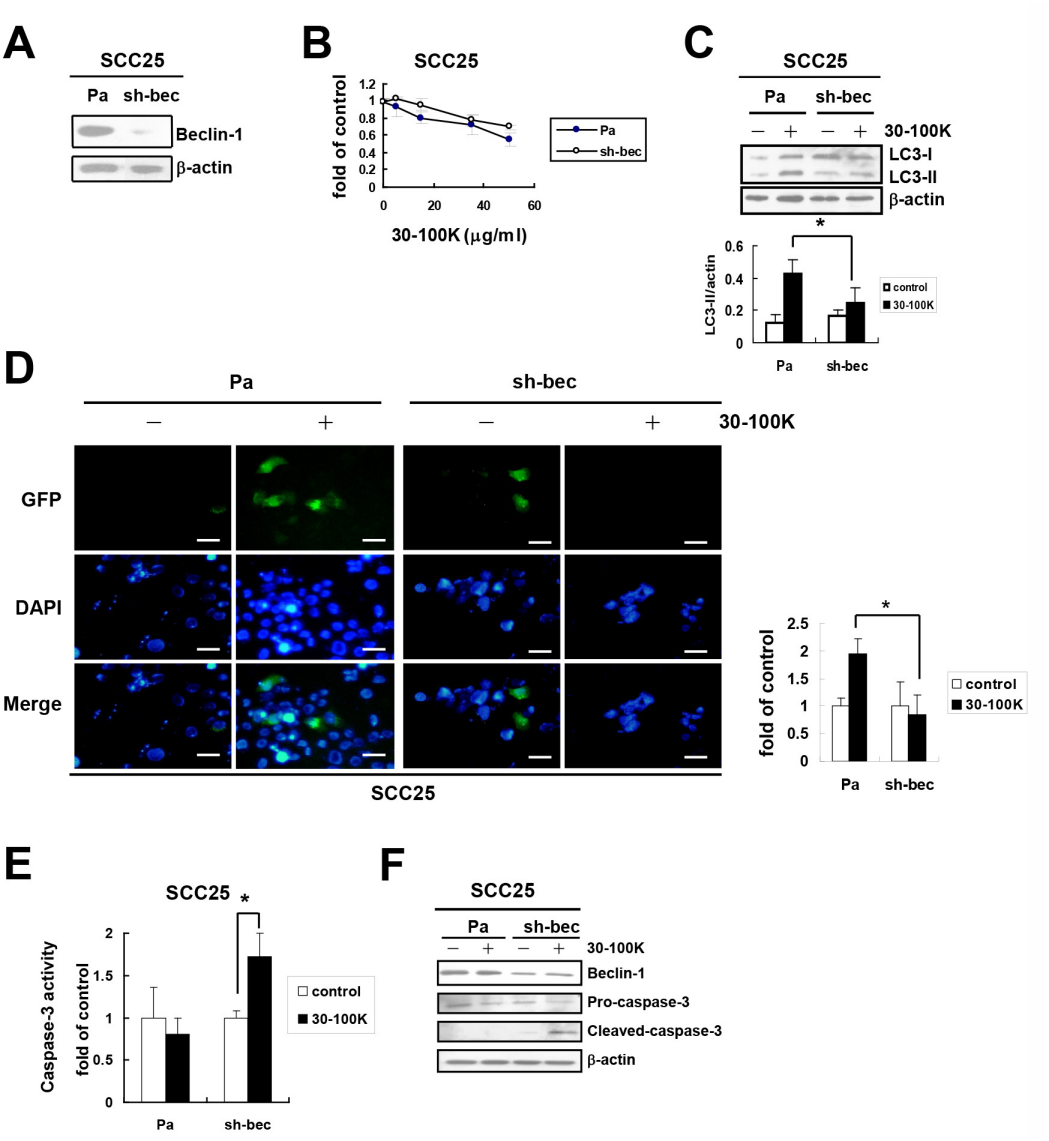
Beclin 1 knockdown inhibits AIA and activates caspases-3 in SCC25 cells. (A) Beclin 1 expression in SCC25 was inhibited as Fig 5 without further cloning. Parental (Pa) and Beclin 1-knocked down (sh-bec) SCC25 cells were subjected to Beclin 1 and β-actin immunoblotting. (B) Viability of Pa and sh-bec cells treated with indicated concentrations of ANE 30–100K (30–100K) was determined and presented as Fig 5B. (C) Induction of LC3-II protein by 30–100K in these two cells was analyzed and presented as Fig 5C. (D) Induction of LC3-GFP puncta by 30–100K in these two cells was analyzed and presented as Fig 5D. (E) Caspase-3 activity in Pa and sh-bec SCC25 cells treated with 30–100K (7.5 μg/ml) for 24 hours were assessed and presented as Fig 5E. (F) Lysates of Pa and sh-bec SCC25 cells treated with or without 30–100K (7.5 μg/ml) for 24 hours were immunoblotted with Beclin 1, caspase-3, and β-actin antibodies. *P < 0.05, **P < 0.01.

Similar effects of Beclin 1 knockdown on AIA were observed in other OSCC cell lines. Firstly, by using the same methods without further cloning, Beclin 1 expression could be effectively inhibited in tongue carcinoma SCC25 cells (sh-bec, Fig 6A). These cells also exhibited comparable sensitivity to cytotoxic ANE 30–100K as Pa cells (Fig 6B) and generated lower levels of LC3-II (Fig 6C) and percentage of puncta-containing cells (Fig 6D) than those of Pa cells after ANE 30–100K stimulation. ANE 30–100K also stimulated caspase-3 activation (Fig 6E) and in addition, the cleaved caspase-3 generation (Fig 6F) in these cells. Secondly, Beclin 1 inhibition in another tongue carcinoma SCC15 also resulted in similar responses to ANE 30–100K as non-infected Pa cells (S4 Fig). These results indicated that Beclin 1 may be another mediator of AIA in different OSCC cells, and ANE 30–100K may activate the apoptotic program when the autophagy machinery such as Beclin 1 is inhibited.

### AMPK is the upstream activator of ERK along ANE 30–100K-mediated pathway

**Fig 7:**
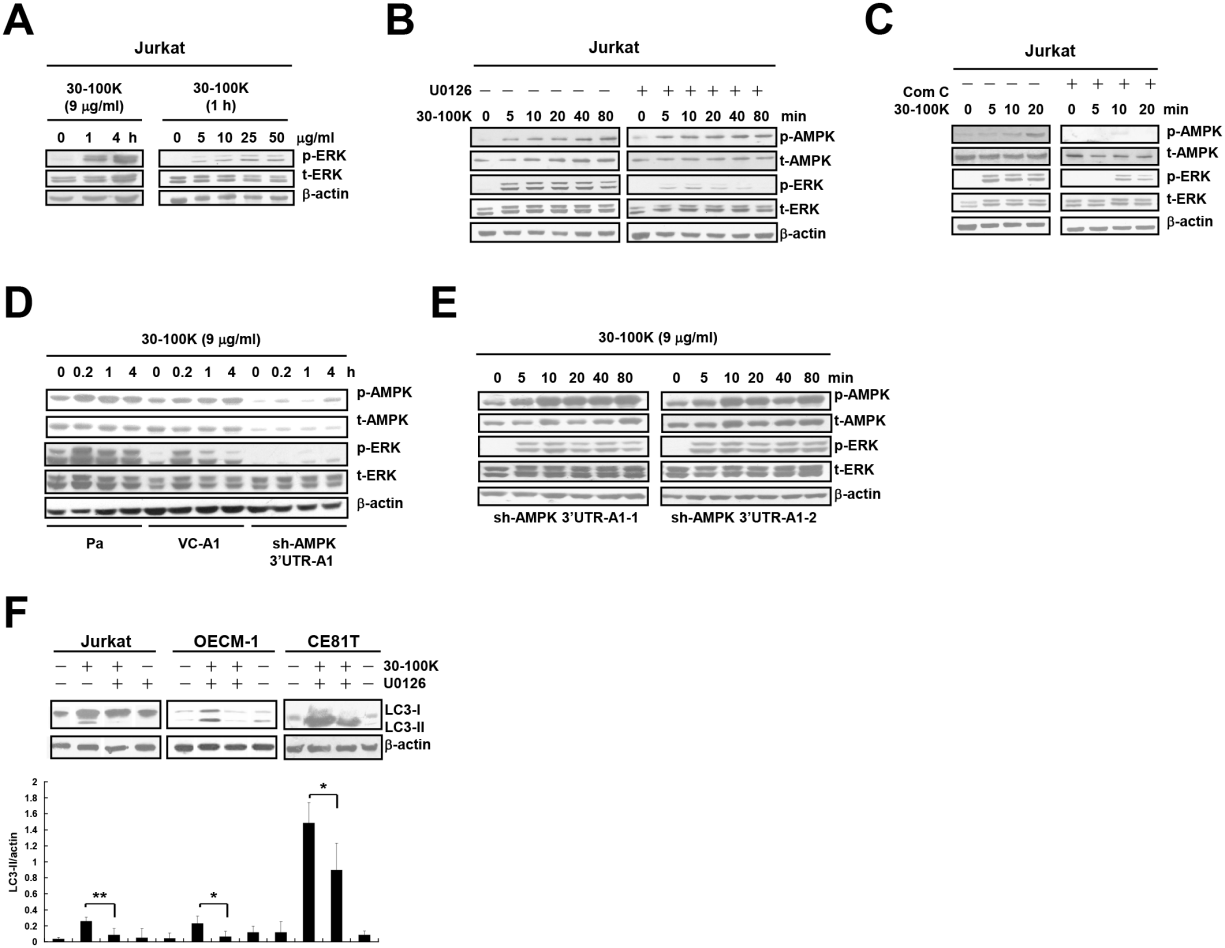
AMPK is an upstream positive regulator of ERK along AIA pathway of Jurkat T cells. (A) Jurkat T cells treated with the indicated periods of ANE 30–100K (30–100K) (9 μg/ml) or with the indicated concentrations of 30–100K for 1 hour were subjected to immunoblotting with phospho-ERK1/2 (p-ERK), total-ERK1/2 (t-ERK), and β-actin antibodies. (B) Cells treated with the indicated periods of 30–100K (9 μg/ml) in the presence or absence of U0126 (10 μM) were subjected to immunoblotting with phospho-AMPKα-Thr^**172**^ (p-AMPK), total AMPK (t-AMPK), p-ERK, t-ERK, and β-actin antibodies. (C) The same experiment was performed as (B), except that U0126 was substituted by compound C (Com C, 5 μM). (D) Lysates of parental (Pa), virus control (VC-A1), and sh-AMPK 3’UTR-A1 cells treated with the indicated periods of 30–100K (9 μg/ml) were subjected to immunoblotting with p-AMPK, t-AMPK, p-ERK, t-ERK, and xx-actin antibodies. (E) The same 30–100K treatment and immunoblotting were performed in sh-AMPK 3’UTR-A1-1 and 3’UTR-A1-2 cells and presented as (D). (F) Lysates of Jurkat T and OECM-1 cells treated with 30–100K (9 and 15 μg/ml, respectively) for the indicated periods in the presence or absence of U0126 (10 μM) were immunoblotted as Fig 1A. Average LC3-II/actin ratios ± SD of 30–100K treatment for 24 hours of both cells were plotted. *P < 0.05, **P < 0.01.

Some signaling mechanisms of ANE 30–100K were also investigated. We demonstrated that ANE 30–100K induced ERK phosphorylation in both time- and concentration-dependent manners in Jurkat T cells (Fig 7A). The MEK inhibitor U0126 inhibited the phosphorylation of ERK but not that of AMPK (Fig 7B), suggesting MEK to be the upstream kinase of ERK but not of AMPK. On the other hand, compound C ameliorated the phosphorylation of both AMPK and ERK, indicating ERK inactivation under AMPK-inhibited conditions (Fig 7C). Furthermore, in contrast to those in Pa and VC-A1 cells, ANE 30–100K-induced ERK phosphorylation was almost abolished in AMPK-knocked down sh-AMPK 3’UTR-A1 cells (Fig 7D). The rescue of AMPK expression in both sh-AMPK 3’UTR-A1-1 and sh-AMPK 3’UTR-A1-2 clones recovered ANE 30–100K-induced ERK phosphorylation (Fig 7E). These data collectively suggest AMPK as an upstream activator of ERK along AIA pathway in Jurkat T cells.

Finally, we also found that U0126 was able to reduce ANE 30–100K-induced LC3-II accumulation in Jurkat T, OECM-1, and CE81T/VGH cells (Fig 7F), indicating the requirement of MEK for AIA.

### Chronic stimulation with ANE 30–100K confers tumors with stronger resistance against serum starvation

We previously showed that chronic treatment of non-cytotoxic ANE or ANE 30–100K resulted in increased chemoresistance through elevated autophagic activity in OECM-1 and Jurkat T cells [35]. Here, we further demonstrated that chronic ANE 30–100K-stimulated multiple myeloma RPMI8226, lymphoma U937, and SCC15 cells also exhibited higher viability and expressed higher LC3-II level under serum-free conditions for 24 hours than their non-stimulated parental cells (S5A and S5B Fig, respectively). Such growth advantage was attenuated by autophagy inhibitors 3-methyladenine (3-MA) and chloroquine (CQ) (S5C Fig). These *in vitro* evidences suggested that AN usage might promote the survival of tumor cells under serum-limited conditions through upregulation of their autophagy activity.

## Discussion

In this study, we verify the requirement of AMPK for AIA in Jurkat T cells but not in OECM-1 cells. On the other hand, Atg5 and Beclin 1 are shown to be AIA mediators in three different cells (OECM-1/CE81T/VGH/Jurkat T and OECM-1/SCC25/SCC-15, respectively). Inhibition of Beclin 1 expression can switch ANE 30–100K-mediated signals from autophagy to apoptosis. We also illustrate that AMPK is an upstream activator of ERK along ANE 30–100K-mediated pathway in Jurkat T cells, and MEK is required for AIA in Jurkat T, OECM-1, and CE81T/VGH cells.

There are four proposed faces of autophagy in cancers [36]. Thus, clarifying the pro-cancer role of autophagy in a specific type of cancer is essential for consideration of autophagy inhibition in cancer therapy. Our previous study revealed that long-term ANE and ANE 30–100K treatment could upregulate autophagy in OECM-1 and Jurkat T cells to increase their tolerance against cisplatin [35]. Here, we further demonstrate that chronic ANE 30–100K stimulation can also increase the survival of three different cancer cells under serum-limited conditions by the same mechanism. We propose that long-term AN usage might render tumors into a particular type of autophagy-addicted context distinct from other types of cancers with frequent downregulation of Beclin 1 and/or LC3 proteins [37–42].

Although AIA utilizes core autophagy mediators like Beclin 1 and Atg5, some differences exist between AIA and conventional autophagy. For example, compared with glucose deprivation (GD)-induced autophagy, ANE 30–100K-induced AMPK activation is necessary for ERK activation in Jurkat T cells (Fig 7), whereas AMPK activation was shown to inhibit ERK in GD-induced autophagy in colon carcinoma HCT116 cells [43]. We also notice that ANE 30–100K and GD treatment resulted in distinct morphological changes in OECM-1 and CE81T/VGH cells (S6 Fig). Moreover, AIA may be different from the three proposed alternative types of autophagy classified by their dependence on Atg5 and sensitivity to 3-MA [24]. The first two types of autophagy induced by apoptotic stimulants such as H_2_O_2_ and resveratrol or by peptidase-resistant peptides are 3-MA resistant [44,20,45], and the third is Atg5/Atg7-independent and sensitive to 3-MA [46]. Although ANE contains small apoptosis inducers like arecoline, our previous study showed that ANE 30–100K itself is not able to activate caspase-3 [27]. Here, we also demonstrate that it can not stimulate detectable DNA ladder (S7 Fig) in OECM-1 cells, suggesting that ANE 30–100K is not an apoptosis inducer and is different from the first two types of alternative autophagy. Furthermore, in addition to the requirement of Atg5 for AIA illustrated in this study, our previously works also verified the attenuation of ANE 30–100K-mediated cell death by 3-MA [28], supporting the exclusion of AIA as the third alternative autophagy. How AIA is different from the molecular mechanism of conventional autophagy is an interesting issue to be followed.

The illusive role of AMPK in autophagy was also observed in another autophagy-inducing compound alternol, purified from the fermentation products of *Alternaria alternata var*. *monosporus*. This compound either inhibits or activates AMPK in different prostate cancer cells leading to differential autophagy responses [47]. Together with our findings of the alternative requirement of AMPK for AIA, it is suggested that targeting this kinase might not be able to achieve effective autophagy inhibition in some tumors.

In summary, our current studies not only provide new action mechanisms of AN ingredient-induced autophagy but also suggest that AN usage may help tumors survive serum-limited conditions through upregulation of their autophagy activity.

## Supporting Information

S1 Fig ANE 30–100K induces autophagic flux in OECM-1 cells and AMPKα-Thr^172^ phosphorylation in CE81T/VGH cells. (A) Immunoblot demonstrating LC3 and β-actin proteins of OECM-1 cells treated with or without ANE 30–100K (30–100K, 15 μg/ml), chloroquine (CQ, 30 μM) and lysosomal inhibitors (LysInh, pepstatin A 10 μg/ml, E64d 10 μg/ml, and leupeptin 10 μg/ml) as indicated. The average LC3-II/actin ratio ± SD from three independent experiments were plotted under each lane. (B) Lysates of CE81T/VGH cells treated with 30–100K (0–9 μg/ml) for 1 hour (left) or 30–100K (9 μg/ml) for 0–24 hours (right) were immunoblotted with p-AMPK, t-AMPK, and β-actin antibodies. Average p-AMPK/t-AMPK ratio ± SD from three independent experiments were plotted under each lane of (C)-(E). *P < 0.05, **P < 0.01, ***P < 0.001.
(TIF)

S2 Fig AIA in OECM-1 cells is less dependent on AMPK. (A) Lysates of OECM-1 cells treated with the indicated concentrations of ANE 30–100K (30–100K) for 1 hour or with 30–100K (15 μg/ml) for the indicated periods were immunoblotted and presented as Fig 1C. (B) Viability of OECM-1 cells treated with the indicated concentrations of 30–100K for 24 hours with or without the pretreatment of compound C (Com C, 5 μM) or STO-609 (250 μM) for 2 hours was analyzed by XTT assay and presented as Fig 5B. (C) AMPK protein levels of parental OECM-1 cells (Pa), virus control (VC-A5) and AMPK-knocked down clones (sh-AMPK CDS-O2, CDS-O5, CDS-O7, CDS-O8, CDS-O9, and CDS-O10) were analyzed as Fig 3A. (D) Viability of Pa, VC-A5, sh-AMPK CDS-O8, CDS-O9, CDS-O10 cells treated with the indicated concentrations of 30–100K for 24 hours was assessed as (B). (E) Lysates of VC-A5 and sh-AMPK CDS-O10 cells treated with the indicated concentrations of 30–100K for 24 hours were immunoblotted and presented as Fig 1A.
(TIF)

S3 Fig Atg5 is required for AIA in CE81T/VGH and Jurkat T cells. (A) With the same strategy in Fig 4A, virus control clones VC-A3 and VC-A4, and Atg5-knocked down sh-atg5 CDS-A3 and CDS-A5 clones of CE81T/VGH were obtained. Lysates of these cells and parental (Pa) cells were immunoblotted as Fig 4A. (B) Viability of Pa and these four cloned cells treated with the indicated concentrations of ANE 30–100K (30–100K) for 24 hours was analyzed and presented as Fig 4C. (C) 30–100K (9 μg/ml)-induced generation of AV in Pa, VC-A3, VC-A4, and sh-atg5-CDS-A5 cells were measured and presented as S3 Fig. (D) Lysates of VC-A4 and sh-atg5 CDS A5 cells treated with or without 30–100K (9 μg/ml) for 24 hours immunoblotted and presented as Fig 1A. (E) Relative Atg5 level in Pa and sh-atg5 Jurkat T cells (transduced with atg5-shRNA-CDS fragment as Fig 4A without further cloning) were analyzed as (A). (F) The sensitivity of Pa and sh-atg5 Jurkat T cells against 30–100K (0, 6, 12 μg/ml) were assayed and presented as Fig 2C. *P < 0.05, **P < 0.01.
(TIF)

S4 Fig Beclin 1 knockdown inhibits AIA and activates caspases-3 in SCC15 cells. By using SCC15 cells, shRNA interference of Beclin 1 (A) and sensitivity against ANE 30–100K (30–100K) (B), as well as induction of LC3-II level (C) and stimulation of caspase-3 activity (D) by 30–100K were identically performed and analyzed as those of SCC25 cells (Fig 6A, 6B, 6C and 6E, respectively). ***P < 0.001.
(TIF)

S5 Fig Chronic stimulation with ANE 30–100K confers tumors with stronger resistance against serum starvation. (A) RPMI8226, U937, and SCC15 cells stimulated with ANE 30–100K (30–100Ks), as well as their non-stimulated parental (Pa) cells were cultured under serum-free (SF) conditions for 24 hours and assessed by XTT. (B) Lysates of the cells in (A) were subjected to immunoblotting with LC3 and β-actin antibodies and data were presented as Fig 1A. (C) Cells cultured in SF medium for 24 hours with or without the pretreatment of 3-MA (1 μM) or CQ (25 μM) were assayed by XTT and presented as Fig 4C. *P < 0.05, **P < 0.01.
(TIF)

S6 Fig Glucose deprivation and ANE 30–100K induce different morphological changes. OECM-1 (A) and CE81T/VGH (B) cells treated with glucose deprivation (GD) or ANE 30–100K (30–100K, 40 and 96 μg/ml, respectively) were photographed after the indicated periods under light microscope. Arrowheads point to the apoptotic-like structures after GD treatment, whereas solid arrows and dotted arrows indicate cells with visible intracellular vesicles and hollow cytoplasm, respectively, after 30–100K treatment. Bar = 10 μm. Firstly, intracellular vesicles became visible in both cells 3 hours after ANE 30–100K treatment but barely visible throughout the entire process of GD treatment. Secondly, most of the GD-treated OECM-1 and CE81T/VGH cells exhibited shrunken morphology in dying cells, followed by the detachment of dead cells from the culture dish. In contrast, ANE 30–100K seemed to trigger enormous degradation of cytosolic materials after the emergence of intracellular vesicles resulting in clearance of cytoplasm before the death of both cells, and most dying or dead cells remained attached to culture dish at the end of treatment (A and B, 12 hours). Finally, only glucose-starved cells generated apoptotic body-like structures in OECM-1 (6 and 12 hours) and CE81T/VGH (3, 6, and 12 hours), which were not observed in ANE 30–100K-treated cells.
(TIF)

S7 Fig Arecoline, but not ANE and ANE 30–100K, induces DNA fragmentation in OECM-1 cells. Small size DNA of OECM-1 cells treated with arecoline (Are, 200 μg/ml), ANE 30–100K (30–100K, 15 μg/ml), or ANE (25 μg/ml) for 24 hours was collected and separated by agarose electrophoresis and photographed under UV light.
(TIF)
