# Supplementary figures and images for: Impacts of Autophagy-Inducing Ingredient of Areca Nut on Tumor Cells
Source: PLoS One. 2015 May 27;10(5):e0128011. doi: 10.1371/journal.pone.0128011 (PMC4445909; doi:10.1371/journal.pone.0128011)

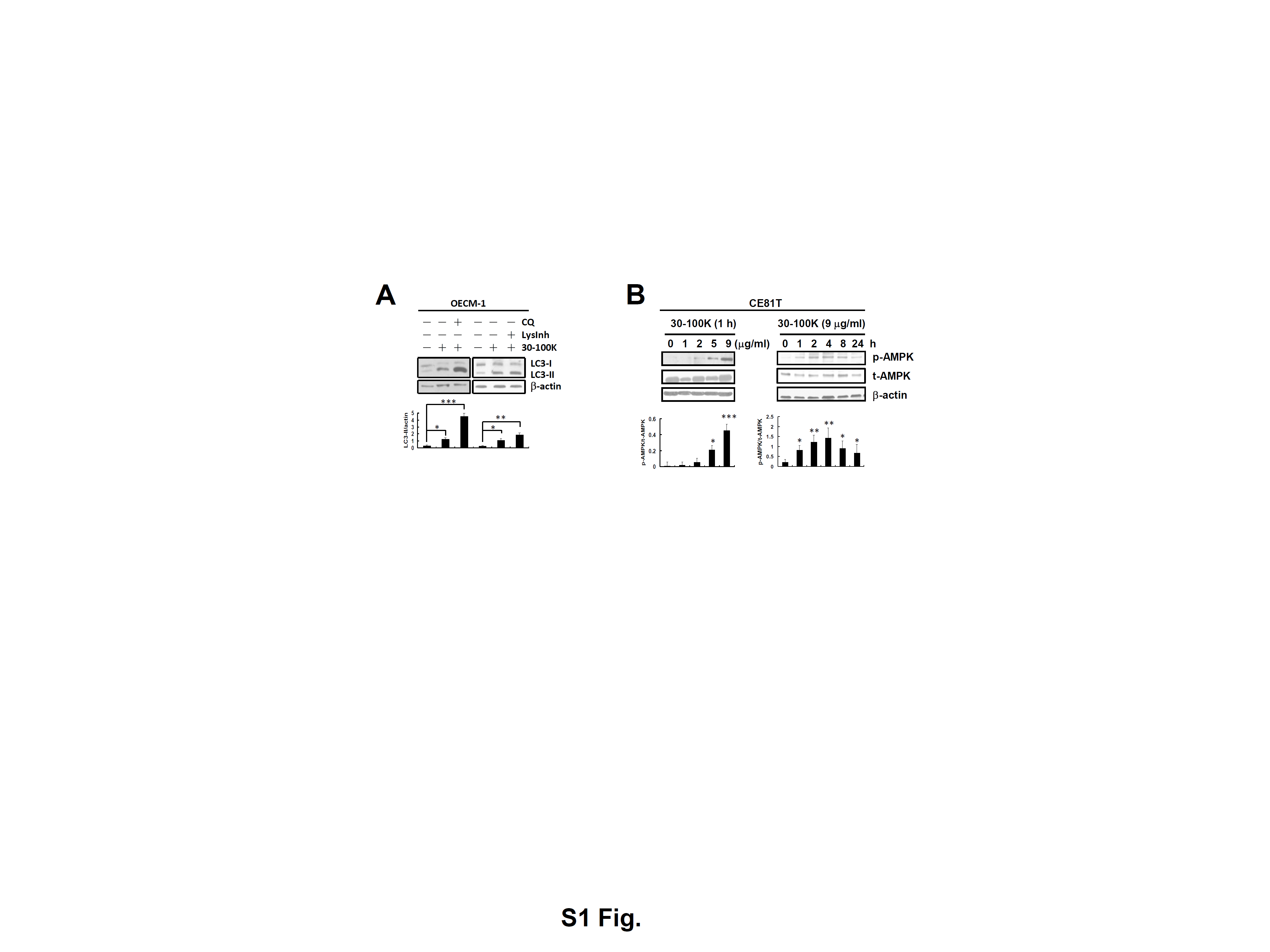

Supplement: S1 Fig — (A) Immunoblot demonstrating LC3 and β-actin proteins of OECM-1 cells treated with or without ANE 30–100K (30–100K, 15 μg/ml), chloroquine (CQ, 30 μM) and lysosomal inhibitors (LysInh, pepstatin A 10 μg/ml, E64d 10 μg/ml, and leupeptin 10 μg/ml) as indicated. The average LC3-II/actin ratio ± SD from three independent experiments were plotted under each lane. (B) Lysates of CE81T/VGH cells treated with 30–100K (0–9 μg/ml) for 1 hour (left) or 30–100K (9 μg/ml) for 0–24 hours (right) were immunoblotted with p-AMPK, t-AMPK, and β-actin antibodies. Average p-AMPK/t-AMPK ratio ± SD from three independent experiments were plotted under each lane of (C)-(E). *P < 0.05, **P < 0.01, ***P < 0.001. (TIF) [file pone.0128011.s001.tif]

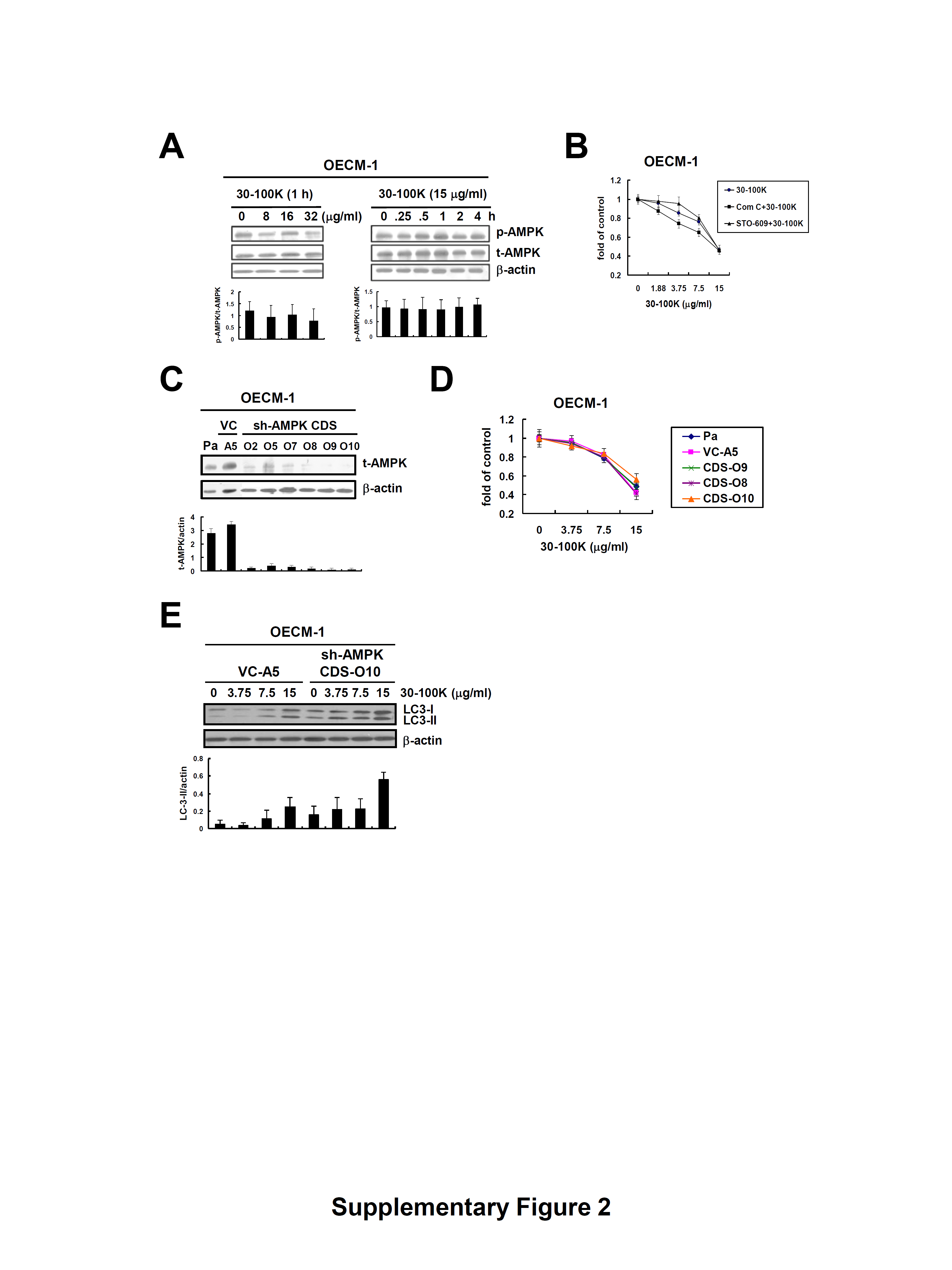

Supplement: S2 Fig — (A) Lysates of OECM-1 cells treated with the indicated concentrations of ANE 30–100K (30–100K) for 1 hour or with 30–100K (15 μg/ml) for the indicated periods were immunoblotted and presented as Fig 1C. (B) Viability of OECM-1 cells treated with the indicated concentrations of 30–100K for 24 hours with or without the pretreatment of compound C (Com C, 5 μM) or STO-609 (250 μM) for 2 hours was analyzed by XTT assay and presented as Fig 5B. (C) AMPK protein levels of parental OECM-1 cells (Pa), virus control (VC-A5) and AMPK-knocked down clones (sh-AMPK CDS-O2, CDS-O5, CDS-O7, CDS-O8, CDS-O9, and CDS-O10) were analyzed as Fig 3A. (D) Viability of Pa, VC-A5, sh-AMPK CDS-O8, CDS-O9, CDS-O10 cells treated with the indicated concentrations of 30–100K for 24 hours was assessed as (B). (E) Lysates of VC-A5 and sh-AMPK CDS-O10 cells treated with the indicated concentrations of 30–100K for 24 hours were immunoblotted and presented as Fig 1A. (TIF) [file pone.0128011.s002.tif]

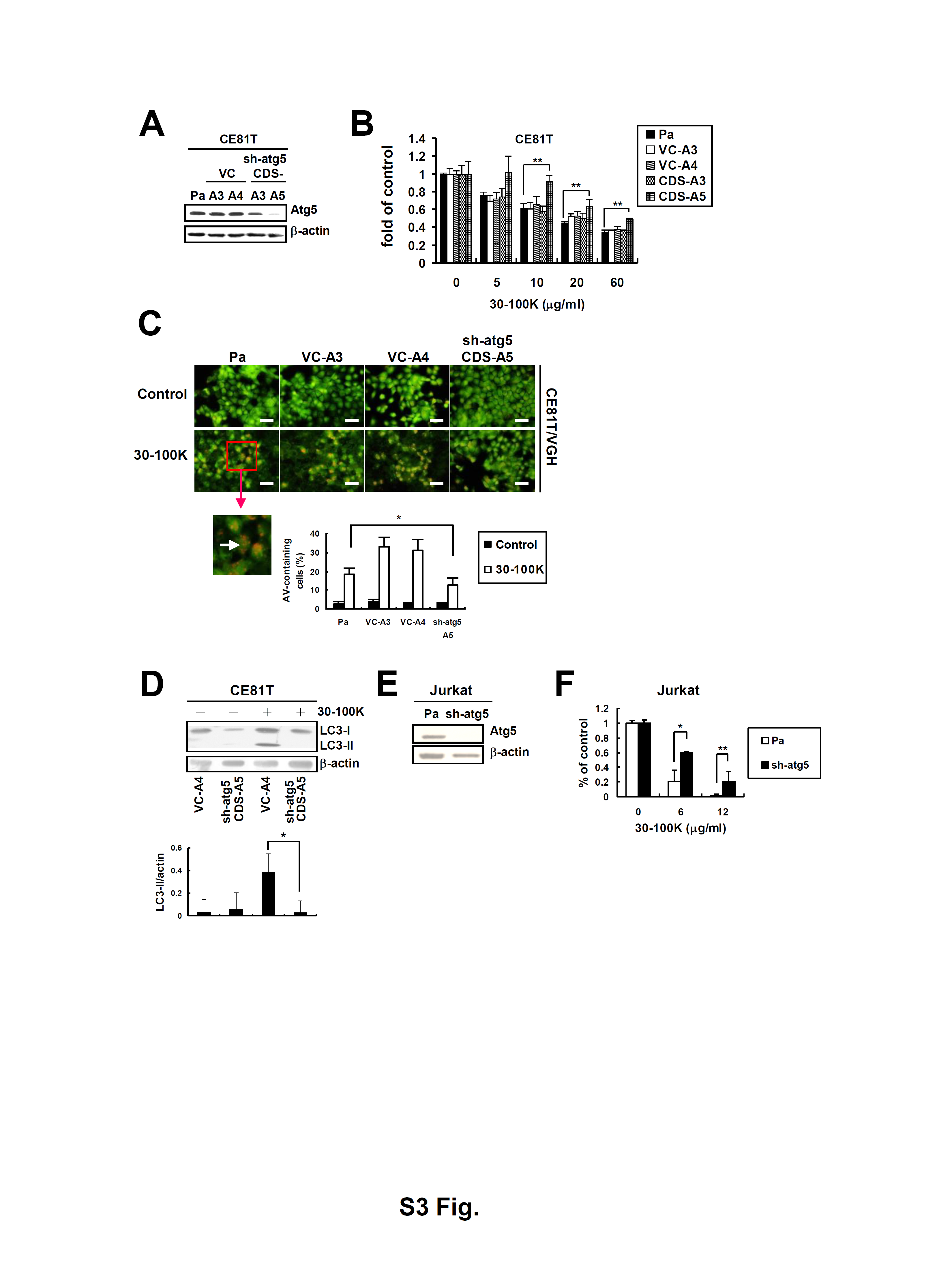

Supplement: S3 Fig — (A) With the same strategy in Fig 4A, virus control clones VC-A3 and VC-A4, and Atg5-knocked down sh-atg5 CDS-A3 and CDS-A5 clones of CE81T/VGH were obtained. Lysates of these cells and parental (Pa) cells were immunoblotted as Fig 4A. (B) Viability of Pa and these four cloned cells treated with the indicated concentrations of ANE 30–100K (30–100K) for 24 hours was analyzed and presented as Fig 4C. (C) 30–100K (9 μg/ml)-induced generation of AV in Pa, VC-A3, VC-A4, and sh-atg5-CDS-A5 cells were measured and presented as S3 Fig. (D) Lysates of VC-A4 and sh-atg5 CDS A5 cells treated with or without 30–100K (9 μg/ml) for 24 hours immunoblotted and presented as Fig 1A. (E) Relative Atg5 level in Pa and sh-atg5 Jurkat T cells (transduced with atg5-shRNA-CDS fragment as Fig 4A without further cloning) were analyzed as (A). (F) The sensitivity of Pa and sh-atg5 Jurkat T cells against 30–100K (0, 6, 12 μg/ml) were assayed and presented as Fig 2C. *P < 0.05, **P < 0.01. (TIF) [file pone.0128011.s003.tif]

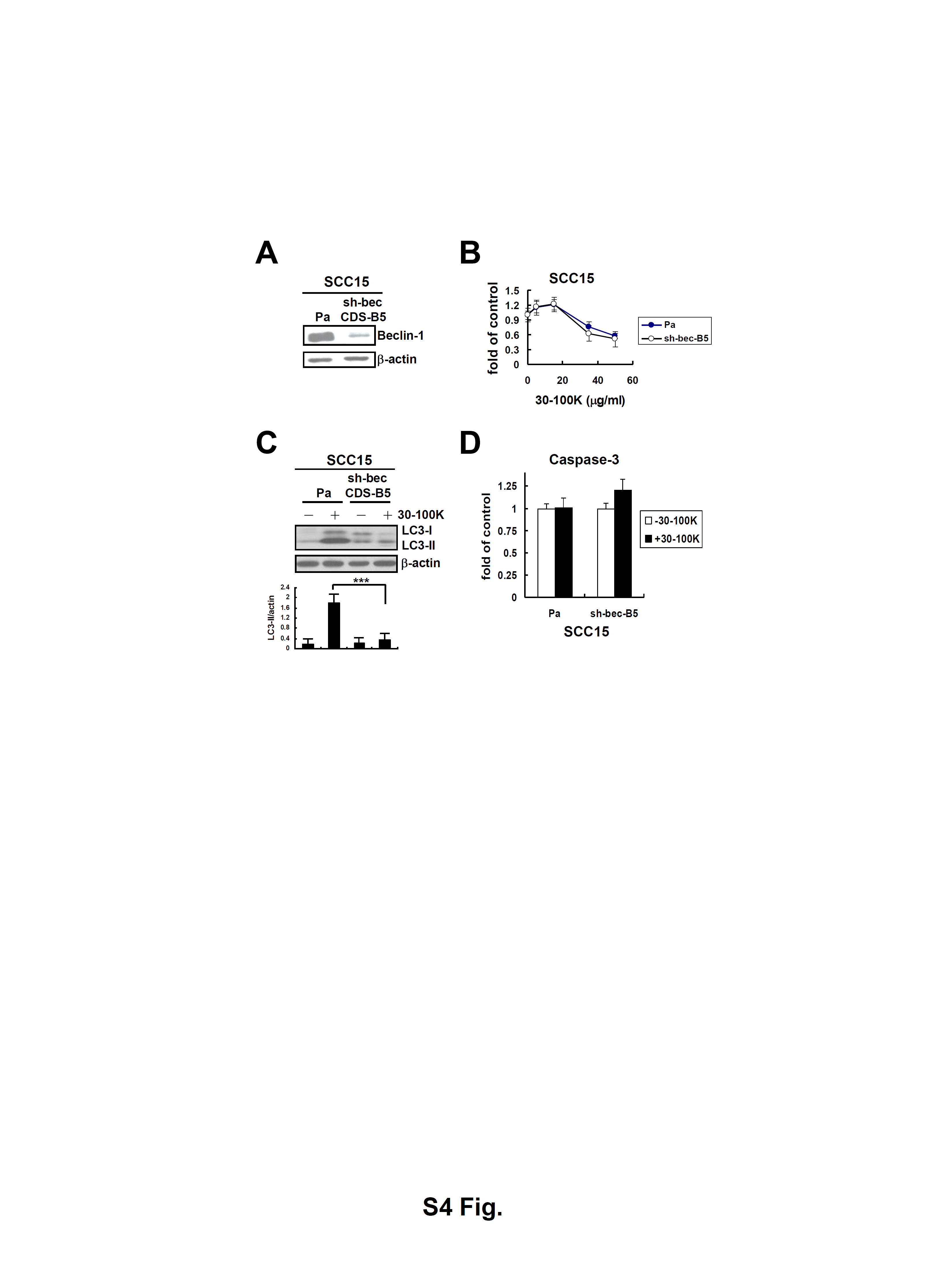

Supplement: S4 Fig — By using SCC15 cells, shRNA interference of Beclin 1 (A) and sensitivity against ANE 30–100K (30–100K) (B), as well as induction of LC3-II level (C) and stimulation of caspase-3 activity (D) by 30–100K were identically performed and analyzed as those of SCC25 cells (Fig 6A, 6B, 6C and 6E, respectively). ***P < 0.001. (TIF) [file pone.0128011.s004.tif]

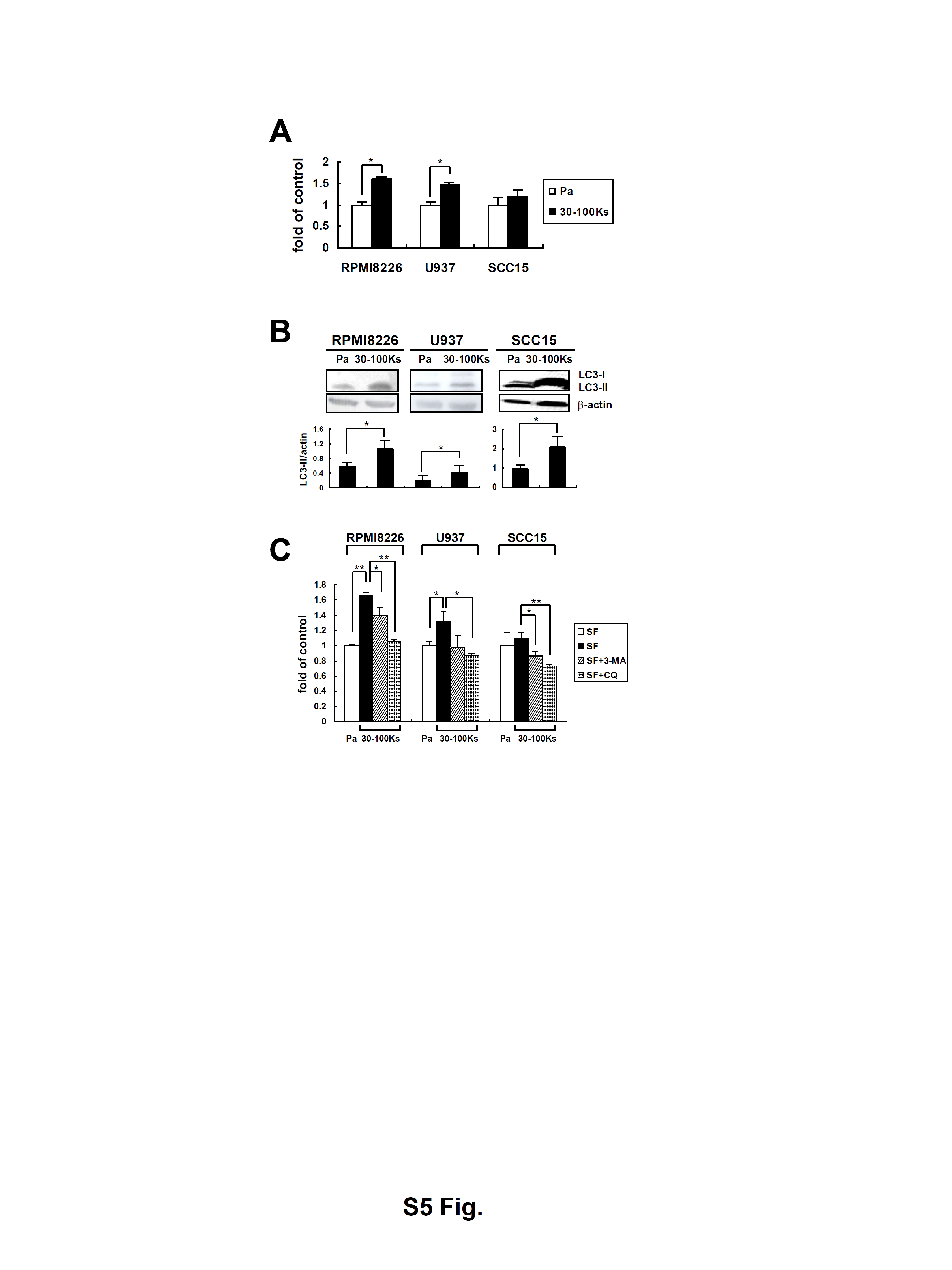

Supplement: S5 Fig — (A) RPMI8226, U937, and SCC15 cells stimulated with ANE 30–100K (30–100Ks), as well as their non-stimulated parental (Pa) cells were cultured under serum-free (SF) conditions for 24 hours and assessed by XTT. (B) Lysates of the cells in (A) were subjected to immunoblotting with LC3 and β-actin antibodies and data were presented as Fig 1A. (C) Cells cultured in SF medium for 24 hours with or without the pretreatment of 3-MA (1 μM) or CQ (25 μM) were assayed by XTT and presented as Fig 4C. *P < 0.05, **P < 0.01. (TIF) [file pone.0128011.s005.tif]

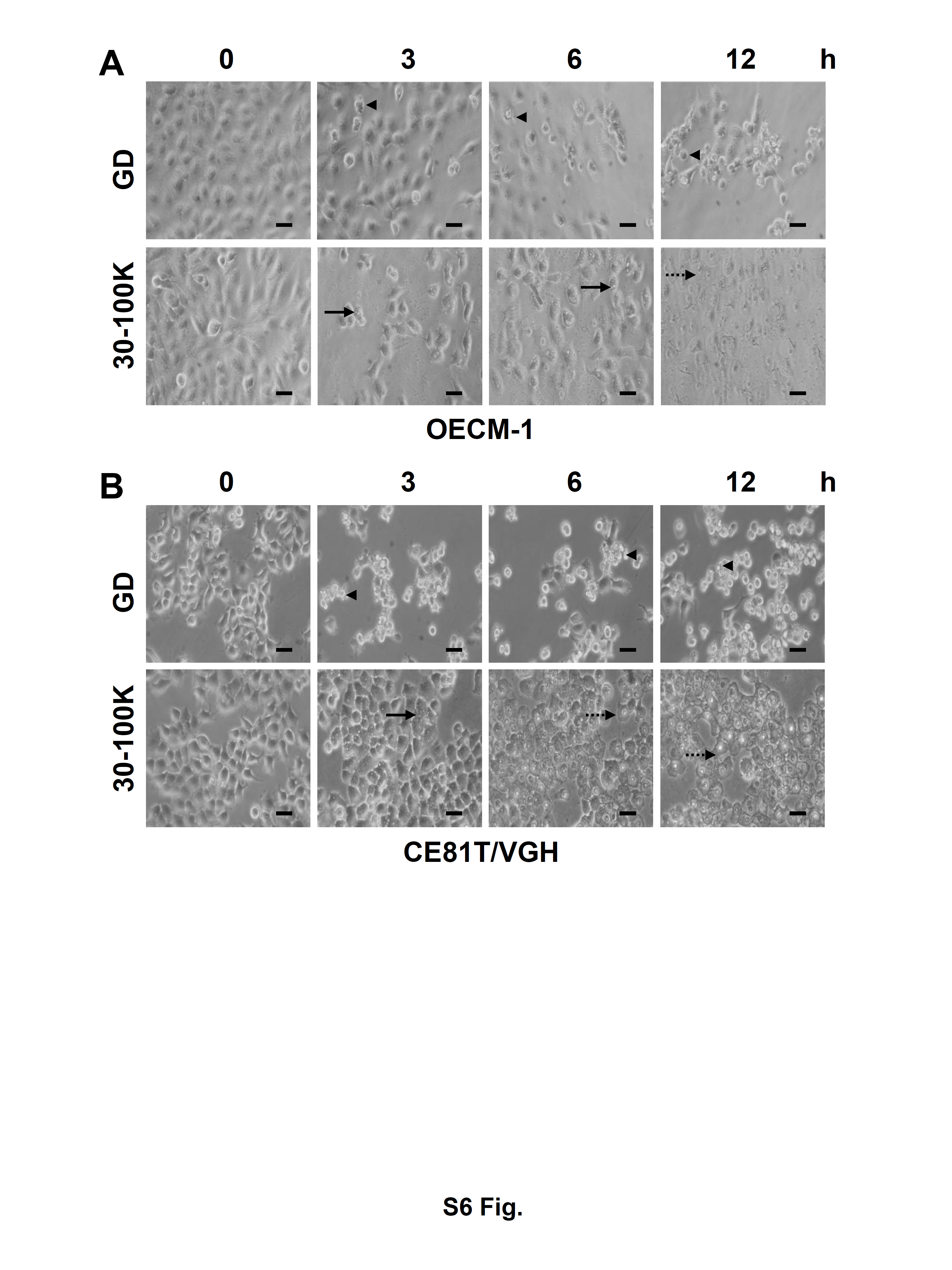

Supplement: S6 Fig — OECM-1 (A) and CE81T/VGH (B) cells treated with glucose deprivation (GD) or ANE 30–100K (30–100K, 40 and 96 μg/ml, respectively) were photographed after the indicated periods under light microscope. Arrowheads point to the apoptotic-like structures after GD treatment, whereas solid arrows and dotted arrows indicate cells with visible intracellular vesicles and hollow cytoplasm, respectively, after 30–100K treatment. Bar = 10 μm. Firstly, intracellular vesicles became visible in both cells 3 hours after ANE 30–100K treatment but barely visible throughout the entire process of GD treatment. Secondly, most of the GD-treated OECM-1 and CE81T/VGH cells exhibited shrunken morphology in dying cells, followed by the detachment of dead cells from the culture dish. In contrast, ANE 30–100K seemed to trigger enormous degradation of cytosolic materials after the emergence of intracellular vesicles resulting in clearance of cytoplasm before the death of both cells, and most dying or dead cells remained attached to culture dish at the end of treatment (A and B, 12 hours). Finally, only glucose-starved cells generated apoptotic body-like structures in OECM-1 (6 and 12 hours) and CE81T/VGH (3, 6, and 12 hours), which were not observed in ANE 30–100K-treated cells. (TIF) [file pone.0128011.s006.tif]

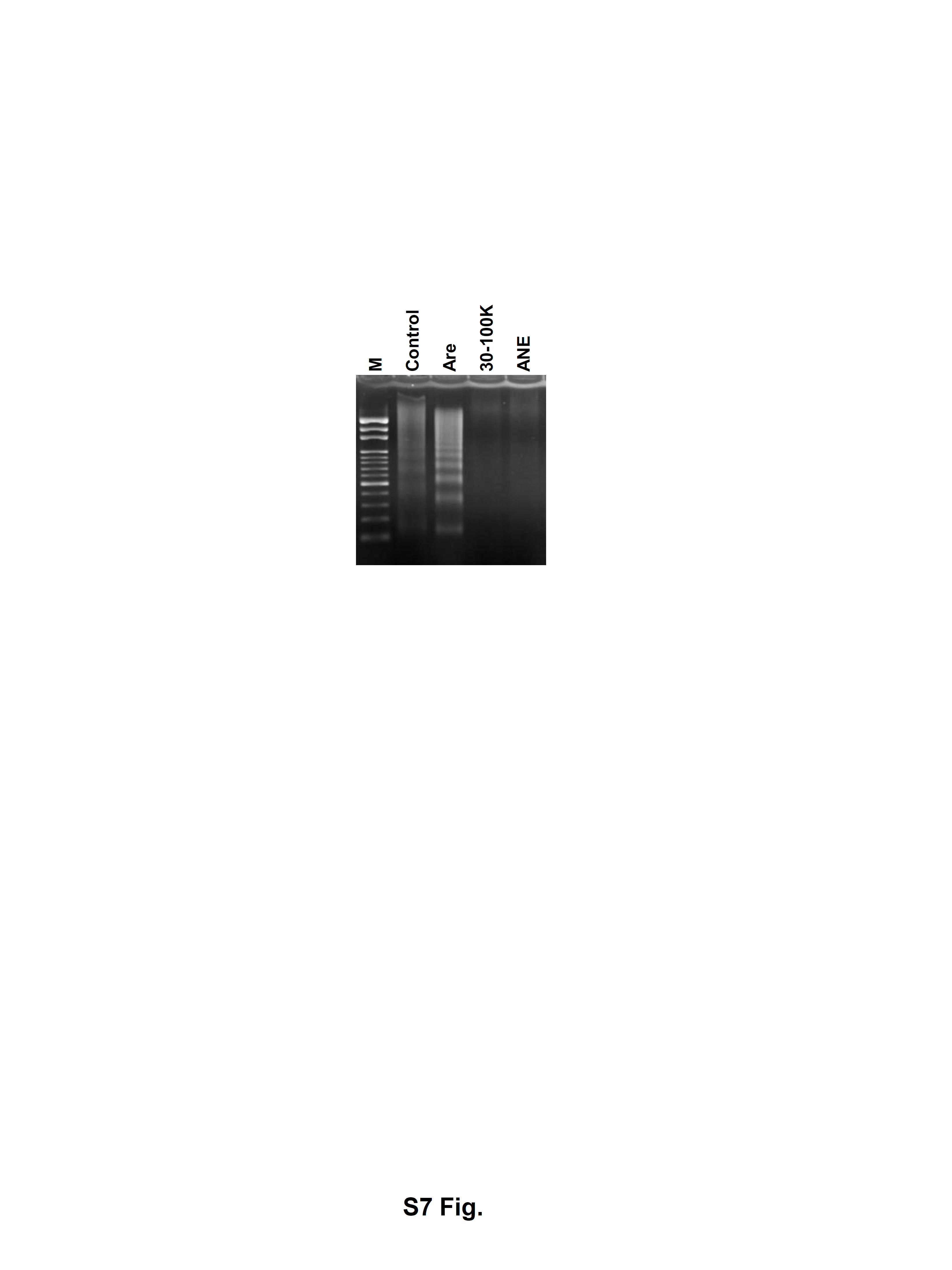

Supplement: S7 Fig — Small size DNA of OECM-1 cells treated with arecoline (Are, 200 μg/ml), ANE 30–100K (30–100K, 15 μg/ml), or ANE (25 μg/ml) for 24 hours was collected and separated by agarose electrophoresis and photographed under UV light. (TIF) [file pone.0128011.s007.tif]
